# Tau Phosphorylation as an Adaptive Physiological Response: Implications for the Therapy of Tauopathies

**DOI:** 10.1096/fj.202503803R

**Published:** 2026-02-09

**Authors:** Timothy Daly, Bruno P. Imbimbo

**Affiliations:** ^1^ Bioethics Program, FLACSO Argentina Buenos Aires Tucumán Argentina; ^2^ Science Norms Democracy, Sorbonne Université CNRS UMR 8011 Paris France; ^3^ Research & Development, Chiesi Farmaceutici Parma Italy

## Abstract

Recent evidence demonstrates that tau phosphorylation, traditionally viewed as a hallmark of neurodegeneration, also occurs in completely reversible physiological contexts such as mammalian hibernation and human neonatal development. These findings challenge the classical protein‐centric “proteinopathy” model of Alzheimer's disease (AD) and other tauopathies. Instead, we propose that phosphorylated tau (p‐tau) functions as an adaptive molecular response to metabolic or neuronal activity shifts, and that tauopathies such as AD represent a failure of broader mechanisms that normally restore tau protein homeostasis. Therapeutic strategies should focus on restoring tau protein homeostasis and functionality rather than simply removing phosphorylated species. To achieve this, we discuss one possible therapeutic strategy: dismantling aggregated tau species.

## Graphical abstract

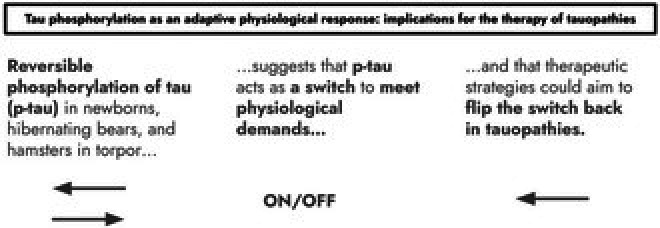

Drawing on recent studies suggesting that tau phosphorylation is reversible, therapies for tauopathies could enhance reversibility, for example, by dismantling aggregated tau species.

## 1 Introduction

The dominant theoretical framework in neurodegeneration research since the 1990s has assumed that abnormal protein accumulation in the brain is intrinsically pathogenic. This theory, known as “proteinopathy”, considers that signature proteins that accumulate in neurodegenerative diseases are also the cause of the disease itself [1]. The therapeutic consequence of this theory is that targeting the underlying proteinopathy will result in disease‐modifying treatments [2].

Alzheimer's disease (AD) is the most common neurodegenerative disease and exhibits a dual proteinopathy: its signature proteins are amyloid‐beta and phosphorylated tau proteins. As a dual proteinopathy, decades‐long debates have raged about “which comes first”. The consensus that emerged from these debates in the 2010s was in line with the so‐called “amyloid cascade hypothesis” according to which amyloid‐beta was the “trigger” and tau the “bullet” of AD degeneration [3]. According to this interpretation, amyloid comes first, but tau is more proximally related to neurodegeneration and likely to be its effector: tau is more strongly correlated with symptom severity than A*β*. More generally, the “tauopathies” are a class of at least 26 different neurodegenerative diseases associated with dementia and parkinsonism, and are characterized by neuronal and/or glial tau‐positive inclusions [4]. Thus, the scientific community has long considered that there are good reasons to believe that tau protein is causally involved in neurodegeneration and has elaborated different mechanistic hypotheses for its putative pathogenicity in tauopathies including AD [5, 6].

However, targeting tau protein has never resulted in a disease‐modifying therapy for AD or any other tauopathy [7]. This can be understood in the broader context of the therapeutic difficulties of targeting signature proteins in neurodegenerative diseases. Moreover, most of the study of tau phosphorylation has focused on its relationship with the symptomatic expression of neurodegenerative syndromes, whereas the question of tau's origins and physiological functions has been under‐studied [8]. Krstic and Knuesel [9] argue that signature proteins in neurodegeneration are like the problem of airbags in car accidents: if we don't know how cars work, we might assume that airbags are killing people in car accidents. This is obviously an incorrect interpretation of causality. Turner [10] similarly argues that the presence of protein aggregates in brain tissues of people who die with neurodegenerative diseases does not mean that those aggregates are the cause: our inferences might suffer from “survivorship bias” because cells that don't develop those aggregates die. These reflections on the complexity of protein function and dysfunction in neurodegeneration set the stage for our discussion of tau protein.

Several recent studies add to a growing body of evidence suggesting that the pathogenic theory of tau phosphorylation may be, physiologically speaking, overly simplistic. We focus on two of those studies in this piece and use them to argue in favor of novel therapeutic approaches for tauopathies.

**FIGURE 1:**
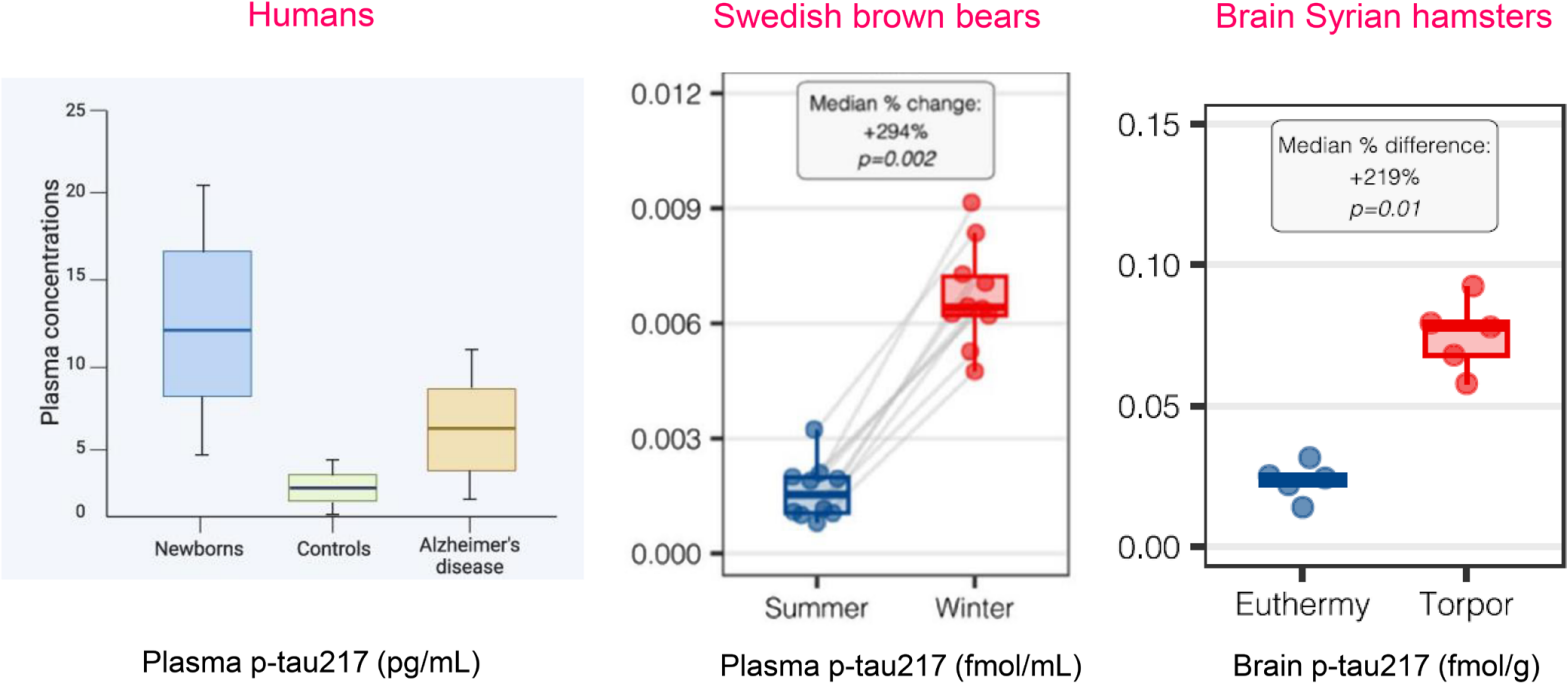
Brain and plasma concentrations of p‐tau217 across species. Plasma levels of p‐tau217 shown for humans (normal neonates, normal adults and subjects with Alzheimer disease, left panel) and Swedish brown bears (during summer and winter hibernation, central panel). Brain levels are depicted for Syrian hamsters (during normal thermal conditions and during refrigeration). “Humans” adapted from Gonzalez‐Ortiz et al. *Brain Commun* 2025 Jun 7;7(3):Fcaf221, “Swedish Brown bears” and “Brain Syrian hamsters” from Brum et al. *Acta neuropathol*. 2025 Sep 29;150(1):36. The re‐use of the content from both of these articles is permitted by a Creative Commons Attribution 4.0 International License https://creativecommons.org/licenses/by/4.0/.

Recent studies show that phosphorylated tau (p‐tau) elevations can occur in physiological settings. Brum et al. reported that tau hyperphosphorylation occurs during mammalian hibernation, a condition marked by profound metabolic and synaptic downscaling, and is fully reversed upon arousal [11]. Their report provides compelling evidence that tau phosphorylation, long regarded solely as a hallmark of neurodegeneration in Alzheimer's disease, occurs in fully reversible physiological contexts (Figure 1). Their study coincides with recent findings from Gonzalez‐Ortiz et al. that demonstrated that reversible plasma phosphorylated tau‐217 (p‐tau217) levels are strikingly elevated in healthy newborn humans, exceeding concentrations observed in patients with AD or in healthy adults across the lifespan [12] (Figure 1). Together, these reports suggest that tau phosphorylation is not an intrinsically pathogenic event but may serve adaptive functions in response to heightened or suppressed neuronal activity. These findings will lead us to argue that tau restoration rather than removal is likely to be a more appropriate therapeutic approach in tauopathies.

## 2 Tau Expression Beyond the Brain

Tau protein and its phosphorylation occur predominantly in the brain. However, several biological sources outside the central nervous system can also contribute to circulating tau, and these extracerebral sources may significantly influence plasma tau concentrations. Structural and single‐cell transcriptomic analyses demonstrate that tau is expressed in multiple lung cell types, including aerocytes and general capillary endothelial cells. Gillich et al. [13] identified these specialized endothelial populations, and Balczon et al. [14] showed that the gene *MAPT* is expressed in lung endothelium and contributes to microtubule stability in this tissue. These observations support the view that circulating tau, particularly in newborns, may originate in part from the lung, which receives the entirety of the cardiac output.

## 3 Physiological Role of Phosphorylated‐Tau

In hibernating brown bears and golden hamsters, plasma and brain levels of p‐tau181, p‐tau217, p‐tau231, and other phospho‐epitopes increased by up to 300%–400%, without evidence of tau aggregation or microtubule‐binding region fragmentation. Upon return to euthermia, these changes normalized [11]. Similarly, in human newborns, elevated levels of p‐tau217 correspond to intense synaptic plasticity and network remodeling that characterize the perinatal brain, suggesting that p‐tau217 might mediate structural and functional adaptation during neuronal hyperactivity [12]. Thus, both extremes of neural activity, that is, neonatal hyperactivity and hibernation‐associated hypoactivity, elicit reversible tau phosphorylation, implying that p‐tau formation is a physiological mechanism of neuronal adaptation.

The studies by Brum et al. and Gonzalez‐Ortiz et al. converge on the common principle that tau phosphorylation is an intrinsic component of brain plasticity that becomes maladaptive only when the ability to reverse it (what we call regulatory reversibility) is lost. During hibernation, hypometabolism induces reversible synaptic regression and p‐tau elevation that protect neurons from energetic stress. In newborns, elevated p‐tau217 accompanies synaptogenesis and circuit specialization. In AD, chronic metabolic and synaptic stress may trigger the same molecular pathway, but persistent activation may prohibit regulatory reversibility. Thus, rather than being a hallmark of neurodegeneration, p‐tau may signify the brain's attempt to maintain structural integrity under changing energetic or functional constraints.

This reinterpretation carries implications for biomarker research and therapeutic strategies. The widespread adoption of plasma p‐tau assays as diagnostic tools for AD presumes that elevated levels unequivocally reflect a pathological state. However, physiological states such as hibernation, intense neuronal activity, or neurodevelopment can also produce marked increases in circulating p‐tau. Distinguishing between adaptive and pathological states of tau will require integrating p‐tau measures with other information—in particular, cognitive and/or functional state [15].

Moreover, mechanistic parallels between neonatal and hibernation‐related tau phosphorylation raise intriguing questions about shared molecular regulators such as changes in kinase and phosphatase balance, and modulation of heat‐shock and stress‐response pathways. Identifying potential upstream signals, for example, AMPK, GSK‐3*β*, or MAPK cascades that govern these reversible transitions, could reveal how neurons toggle between plasticity and pathology.

Conceptually, viewing p‐tau as a physiological response to brain stimuli reframes tauopathies as disorders of failed reversibility. That is, what is lost is tau protein homeostasis or “proteostasis,” that is, cell‐level control over “the concentration, conformation, binding interactions and location of individual proteins” [16]. Loss of proteostasis is common across neurodegenerative diseases [10]. The dominant interpretation of this loss is that it is caused by toxic protein aggregates, but evidence also supports the notion that proteostasis deficits might occur before protein aggregation [17]. We adopt this point of view of protein homeostasis rather than adopting a pathogenic‐only view of protein aggregation of tau protein.

The pathological accumulation of phosphorylated tau might represent the residue of a once‐adaptive response that becomes self‐perpetuating when cellular energy balance and clearance mechanisms decline due to age‐related vulnerabilities affecting cells' ability to maintain proteostasis [10]. This model aligns with the evolutionary logic that the same molecular programs supporting plasticity and survival during development and stress are those that, when dysregulated by age, drive degeneration [18]. Rust [19] suggests that it is the brain's adaptability across the life course that makes it vulnerable to age‐related degeneration.

## 4 Reversibility of Phosphorylated‐Tau

We challenge the conventional binary view of tau phosphorylation as either “normal” or “pathological.” Phosphorylation is rather a flexible molecular switch that adjusts microtubule dynamics and synaptic organization according to metabolic and electrical demands. The numerous phosphorylation sites (> 50 identified) of tau allow rapid modulation of its microtubule affinity, controlling cytoskeletal remodeling during axonal transport, neurite extension, and synapse formation. The physiological triggers of tau phosphorylation are diverse. Under normal conditions, phosphorylation helps restrict tau to axons and facilitates organelle trafficking. The reversible hyperphosphorylation of tau in hibernation, as documented by Brum et al. resembles the transient phosphorylation of other cytoskeletal proteins that modulate neuronal structure during activity‐dependent plasticity. Similarly, the transiently elevated p‐tau217 in neonates described by Gonzalez‐Ortiz et al. may reflect a developmental requirement for cytoskeletal remodeling rather than a prodromal sign of pathology.

In the context of AD, therefore, increased p‐tau217 may not initially be pathological but rather compensatory. In support of this notion, neurons forming neurofibrillary tangles often survive longer than their non‐tangle‐bearing counterparts, as shown by longitudinal two‐photon microscopy studies in transgenic models [20]. This suggests that phosphorylation and even aggregation could represent late, protective (or failed attempts at protective) adaptations to neuronal injury. What distinguishes physiological from pathological phosphorylation might be its persistence and age‐related failure to restore proteostasis of tau phosphorylation.

## 5 Therapeutic Implications

Therapeutically, note that in Brum et al.'s study, tau fragments derived from the microtubule‐binding region (MTBR), which are associated with tangle aggregation, were not increased. In AD, elevated cerebrospinal fluid and plasma levels of MTBR‐tau reflect brain tangle formation. Specifically, the extended MTBR fragment known as plasma eMTBR‐tau243 serves as a biomarker that increases with the progression of tau tangle pathology, showing strong correlations with tau positron emission tomography (PET) signal and cognitive decline, and could thus be used to help diagnosis and for monitoring treatment efficacy [21].

This pattern suggests that monomeric phosphorylated tau species may play a physiological role, whereas tau aggregation could sequester these functional monomers. Consequently, therapeutic strategies for AD and other tauopathies should not target monomeric phospho‐tau but rather the aggregated tau species, so as to disassemble them and release monomeric phospho‐tau forms, which may restore tau proteostasis and potentially exert beneficial effects. This concept parallels observations with monoclonal anti‐amyloid‐*β* antibodies: treatment with those antibodies which mobilize soluble A*β* monomers from brain plaques were shown to significantly slow cognitive and functional decline [22].

## 6 Testing the Hypothesis of a Compensatory Role of Phosphorylated Tau

Testing the hypothesis that phosphorylated tau represents a compensatory response to physiological or para‐physiological challenges is not straightforward. The first essential step is the precise identification of the biological sources of p‐tau217 and of other phosphorylated tau species. This requires experiments that move beyond the implicit assumption that p‐tau is exclusively of neuronal origin and that it reflects only brain pathology.

A first experimental strategy should focus on source identification. Lung‐specific *MAPT* knockout models can be used to determine whether circulating p‐tau levels decrease in newborn animals when tau expression is selectively removed from lung endothelium and related cell types. In parallel, tau species should be measured in paired samples of pulmonary arterial and pulmonary venous blood. A higher concentration of p‐tau in pulmonary venous versus arterial blood would support a relevant contribution of the lung to circulating p‐tau under defined physiological conditions. Similar approaches could then be extended to other candidate peripheral sources.

A second strategy should examine reversibility and function of phosphorylated tau. Animal models could be exposed to controlled metabolic suppression or to experimental paradigms that enhance neuronal activity. Serial sampling of brain tissue, cerebrospinal fluid, and plasma would allow the characterization of phosphorylation and dephosphorylation cycles over time. The key question is whether p‐tau elevations are tightly coupled to reversible changes in synaptic structure, microtubule dynamics, and network activity. If p‐tau increases parallel adaptive remodeling and then return to baseline when the challenge ends, this would support a compensatory interpretation.

A third strategy should move towards therapeutic testing of the hypothesis of restoring tau proteostasis. As one class of examples, molecules that selectively disassemble tau aggregates without depleting monomeric phosphorylated tau could be evaluated in relevant models of tauopathy. These could include precision antibodies [23]. The main outcomes should include changes in the pool of soluble monomeric p‐tau, neuronal resilience to metabolic or excitotoxic stress, and structural and functional readouts of synaptic integrity. If aggregate disassembly increases adaptive monomeric p‐tau and is associated with improved neuronal survival and function, this would provide strong evidence that at least part of phosphorylated tau signaling is compensatory and not purely degenerative.

Together, these three lines of investigation would address the source of circulating p‐tau, the reversibility and functional meaning of its fluctuations, and the therapeutic consequences of preserving or restoring tau functionality.

## 7 Conclusion

In conclusion, new evidence demonstrates that tau phosphorylation can occur in the absence of neurodegeneration and serves reversible, adaptive purposes. Elevated p‐tau217 may therefore represent a physiological biomarker of neuronal activity and structural remodeling rather than an exclusive indicator of Alzheimer‐type pathology. Future studies should aim to define factors that distinguish beneficial from maladaptive tau phosphorylation as part of a deeper mechanistic understanding [24] of the normal functions of tau protein, which remains insufficient [8]. Harnessing the physiological functions of p‐tau for neuroprotection and reducing pathological trade‐offs is essential for the future of therapeutics.

## Author Contributions

**Timothy Daly and Bruno P. Imbimbo:** contributed equally to all aspects of the manuscript: conceptualization, writing, editing, and validation.

## Funding

The authors have nothing to report.

## Conflicts of Interest

Dr. Timothy Daly declares no conflicts of interest. Dr. Bruno P. Imbimbo is an employee of Chiesi Farmaceutici and is listed as an inventor on several of the company's patents related to anti‐Alzheimer's drugs.

## Data Availability

No new data.
